# Anomalous origin of the left coronary artery from the pulmonary artery (ALCAPA) diagnosed in children and adolescents

**DOI:** 10.1186/s13019-020-01116-z

**Published:** 2020-05-12

**Authors:** Zhang Jinmei, Ling Yunfei, Wang Yue, Qian Yongjun

**Affiliations:** 1 Department of Intensive Care Unit, West China Hospital, Sichuan University, No. 37 GuoXue Xiang, Chengdu, Sichuan 610041 People’s Republic of China; 2 Department of Cardiovascular Surgery, West China Hospital, Sichuan University, No. 37 GuoXue Xiang, Chengdu, Sichuan 610041 People’s Republic of China

**Keywords:** Congenital heart disease, Anomalous origin of the left coronary artery from the pulmonary artery

## Abstract

**Background:**

Anomalous origin of the left coronary artery from the pulmonary artery (ALCAPA) is a rare but potentially fatal congenital coronary anomaly associated with early infant mortality and sudden adult death. By the development or lack of coronary collateral, it can be classified as infantile or adult type. However, even with the compensatory mechanism in adult patients, there is an estimated 80 to 90% incidence of sudden death at the mean age of 35 years.

**Methods:**

We enrolled 9 patients with ALCAPA within the age group 5 to 16 years.

**Results:**

Only one patient developed symptoms (apsychia), whereas other patients were asymptomatic, and there was no evident left ventricular dysfunction found in any of the cases.

**Conclusion:**

With the development of imaging techniques, asymptomatic adult-type ALCAPA patients could be identified and diagnosed in childhood or adolescence. As a potential cause of sudden death, ALCAPA should be surgically repaired soon after the diagnosis.

## Background

Anomalous origin of the left coronary artery from the pulmonary artery (ALCAPA) is a rare but potentially fatal congenital coronary anomaly associated with early infant mortality and sudden adult death. Krause and Brooks reported the first incidences of ALCAPA syndrome in 1865 and 1885, respectively [1, 2]. In 1933, Garland, Bland, and White made the first clinical pathologic correlation, and they also proposed a review of congenital variations in the coronary vessels and a discussion of their embryological background [3, 4]. Garland and associates estimated that ALCAPA is present in 1 out of 300,000 live births or 0.5% of the children with congenital heart disease, and these data were widely cited during past years. With the development of diagnostic techniques, more asymptomatic patients have been diagnosed and treated surgically.

Conventionally, ALCAPA syndrome is classified into two types. There is little or no coronary collateral development in the infantile type of circulation; and after the closure of patent ductus arteriosus, it could lead to severe myocardial ischemia, left ventricle (LV) dysfunction, dilatation, and mitral regurgitation (MR) when the pressure of pulmonary artery (PA) falls [5–7]. The infantile type patients die within weeks to months after birth without surgical correction. About 10 to 15% of the ALCAPA patients are adult-type [7], and their survival is due to a large dominant right coronary artery (RCA) with extensive inter-coronary collaterals, as well as a restrictive opening between the ALCAPA and the PA [3, 8–10]. With ongoing subclinical myocardial ischemia, these patients can be asymptomatic until adulthood. In adult-type ALCAPA patients, there is an estimated 80 to 90% incidence of sudden death at the mean age of 35 years [7, 9, 10].

The diagnosis of ALCAPA in adults is rare [5, 6, 11]. Most ALCAPA patients were diagnosed after the presence of varied clinical presentation and American Heart Association for Adult Congenital Heart Disease treatment guidelines has suggested surgical repair in adult ALCAPA patients regardless of myocardial viability [12]. However, considering the variety of clinical symptoms and cardiac function, it is hard to choose the rational treatment, especially when the patients are asymptomatic and without evident left ventricular dysfunction. We report 9 cases (aged between 5 and 16 years) of ALCAPA, where only one patient developed symptoms (apsychia), and others were asymptomatic, and there was no evident left ventricular dysfunction found in any of the cases.

## Methods

### Study population

We retrospectively studied nine consecutive patients who were diagnosed with ALCAPA in childhood or adolescence from the database of our institution between the years 2010 and 2016. We obtained their clinical and demographic information from patients’ records. The diagnosis of these patients was not established in the infantile period except for who was diagnosed with congenital heart disease when he was hospitalized for “cough”.

## Results

The mean age of the patients at diagnosis was 9.5 years. One five-year-old boy was diagnosed with congenital heart disease when he was hospitalized for “cough” 4 years ago but did not undergo the surgical treatment. One five-year-old girl was diagnosed with endocardial fibroelastosis syndrome in another hospital and went through medical treatment; she was sent to our hospital after the mitral regurgitation worsened. Other patients were diagnosed in our hospital due to the pansystolic murmur at the left sternal border and the apex having been found in the regular physical examination (Table 1).

**Table 1:** ALCAPA syndrome diagnosed in children and adolescents: patient demographics, clinical, imaging data and outcome data

Case	1	2	3	4	5	6	7	8	9
Gender	F	M	M	M	F	F	M	M	M
Age	15	13	5	14	7	5	6	16	5
Presenting symptoms	NO	NO	NO	syncope	NO	No	No	No	No
NYHA Class	I	II	I	I	I	I	I	I	I
Heart Murmur	Yes	Yes	Yes	Yes	Yes	Yes	Yes	Yes	Yes
Abnormal ECG	Yes	–	No	Yes	Yes	Yes	Yes	Yes	Yes
Abnormal CXR	Yes	Yes	Yes	–	Yes	Yes	Yes	No	Yes
MR grade at diagnosis	mild	No	No	mild	severe	severe	moderate	moderate	No
LVEF%	66	64	68	66	65	59	63	70	70
Preoperative arrhythmia	No	No	No	No	No	No	No	No	No
Type of ALCAPA surgery	Takeuchi/VSD closure	N/A	reimplantation	Takeuchi	reimplantation/Mitral repair	reimplantation/Mitral repair	reimplantation	Takeuchi	reimplantation
Follow up to date (years)	7	–	0.5	0.5	3	1	1	2	1
Postoperative arrhythmia	No	–	–	No	No	No	No	No	No
Device implantation	No	–	No	No	No	No	No	Yes	No
CPB Time (min)	145	–	145	150	171	175	76	118	146
Aortic Occlusion Time (min)	103	–	103	116	138	128	45	91	117
Transfusion	Yes	–	Yes	Yes	Yes	Yes	Yes	Yes	Yes

**Fig. 1:**
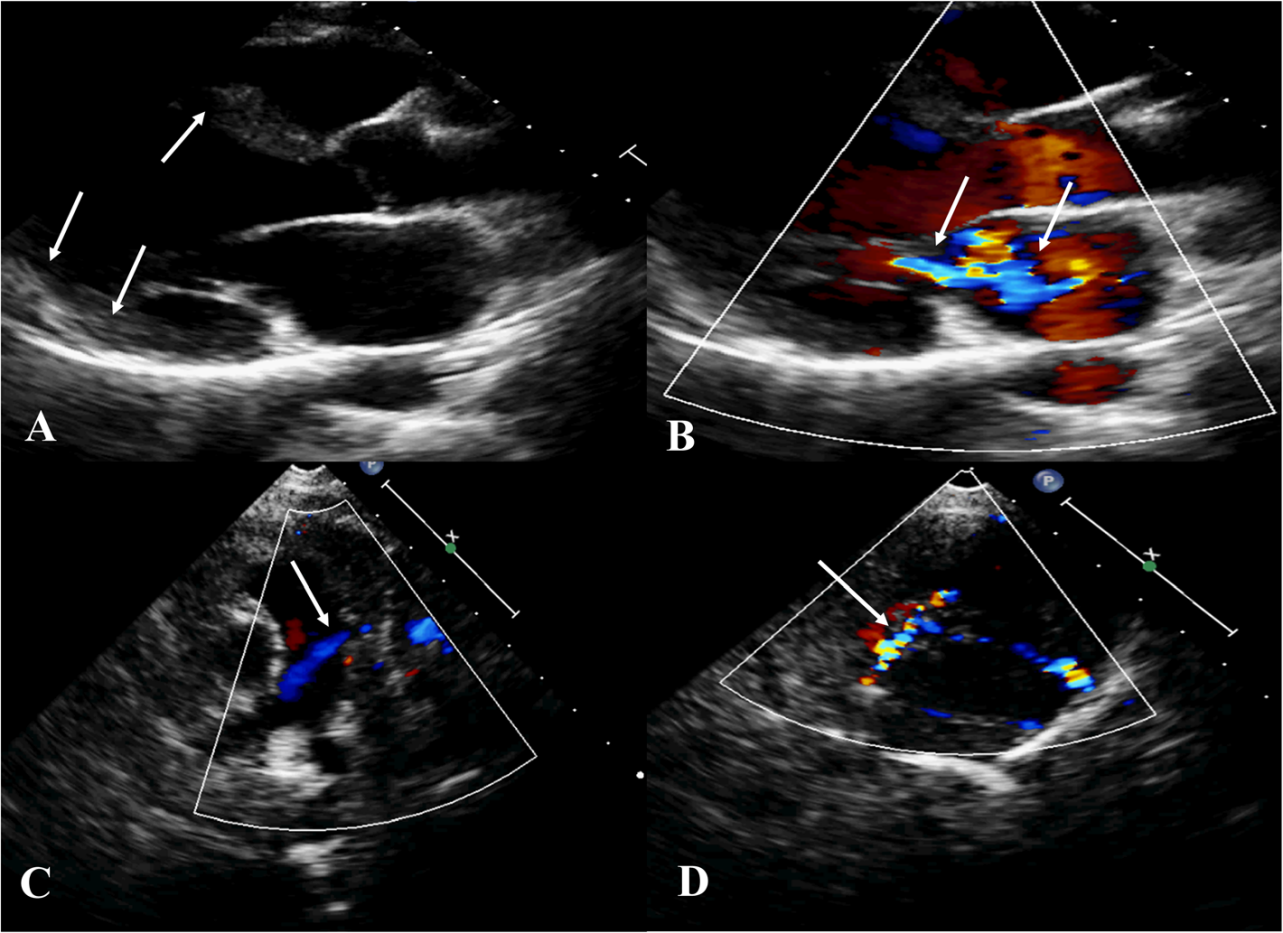
**a** 2D on parasternal long axis view. White arrows show the dilated left ventricle (LV). **b** Color 2D on parasternal long axis view. White arrows show Mitral regurgitation (MR). **c** Color 2D on parasternal short axis view. White arrow shows the retrograde flow from the left coronary artery (LCA) into the pulmonary artery. **d** Color 2D on parasternal short axis view. White arrow shows the increased flow in the intraventricular collateral vessels from the RCA to the LCA

**Fig. 2:**
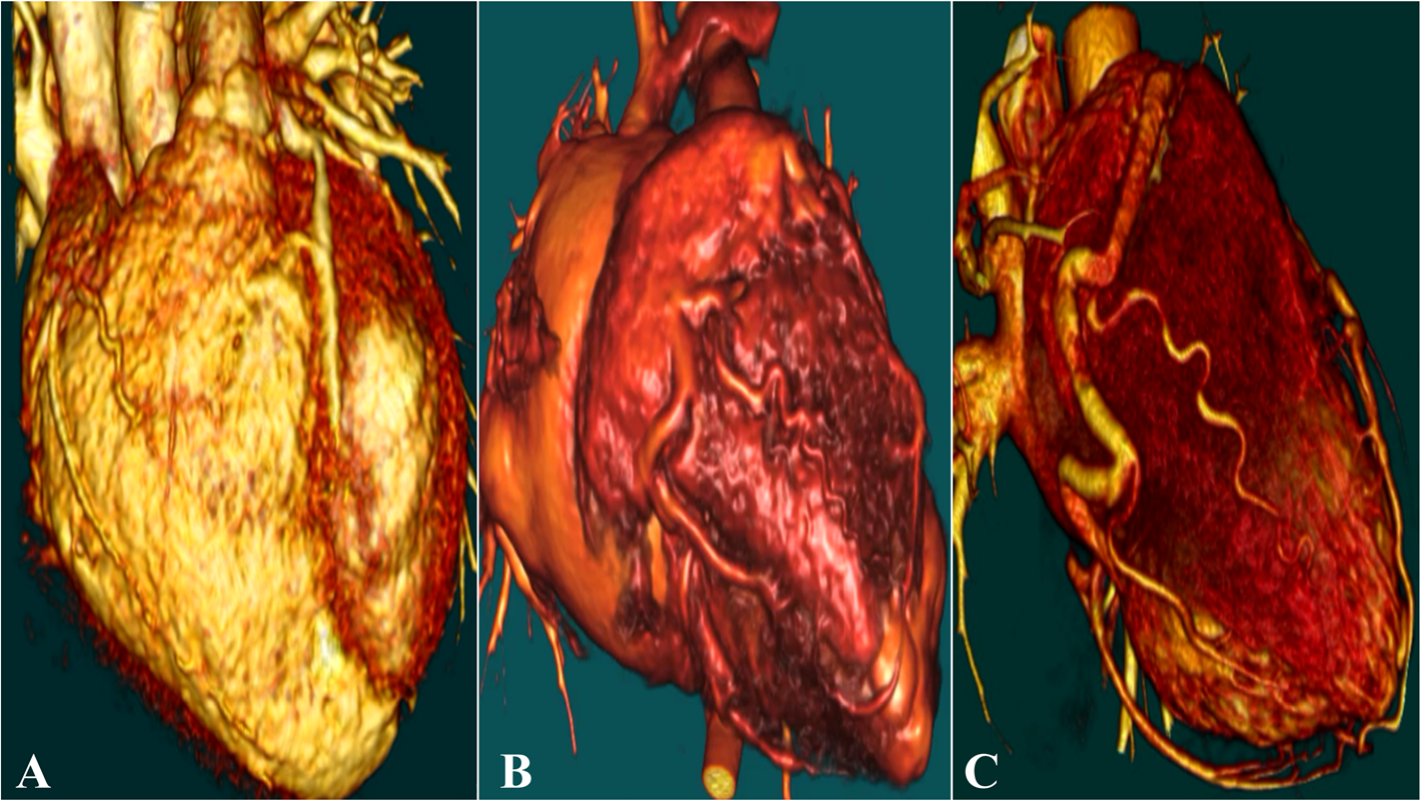
CT coronary angiogram - 3D reconstruction shows different levels of collateral vessels from the right coronary artery (RCA) to the left coronary artery (LCA) in 5-year-old boy (**a**), 7-year-old girl (**b**), 16-year-old boy(**c**)

The pansystolic murmur was found in all the 9 patients. Seven patients (77.8%) had cardiomegaly on CXR; one patient (11.1%) appeared to be normal and another did not go through CXR. ECG changes in the lateral or precordial leads were recorded in 7 (77.8%) patients. Echocardiography could provide an initial diagnosis. All the patients (100%) showed sufficient LV function. MR was confirmed in six patients (66.7%) (Fig. 1) and two of them needed surgery. CT, CMR, or coronary angiography confirmed the diagnosis in all the patients. These methods identified a single large RCA derived from the aorta with extensive LCA-derived collaterals draining into the PA (Fig. 2).

**Fig. 3:**
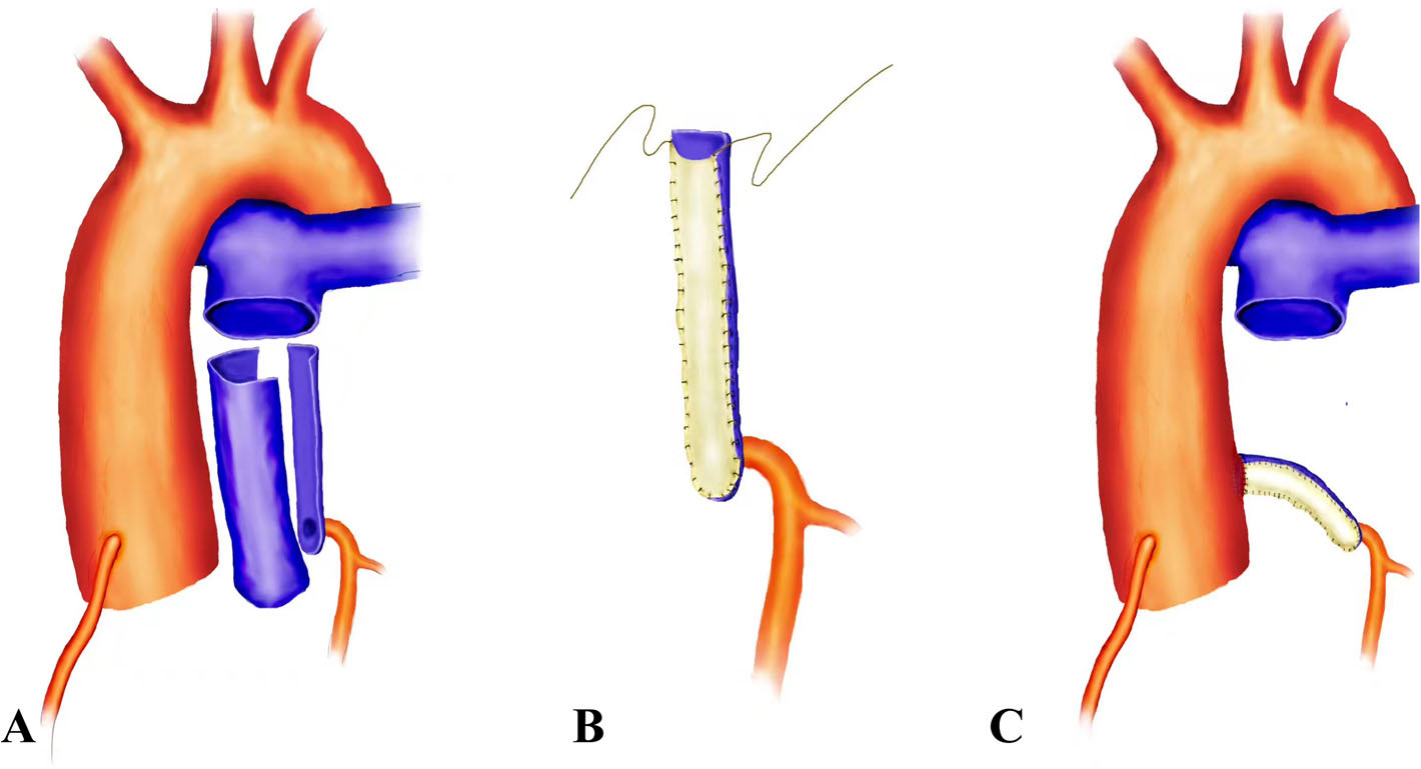
Surgical steps of the re-implantation technique are shown in order. **a** The pulmonary artery is transected just proximal to the bifurcation; one-third circumference of the posterior pulmonary artery is excised including the ALCAPA orifice and the entire sinus, creating an ample-sized autologous flap. **b** By suturing the edges of this flap longitudinally with the autologous pericardial patch, a long rolled conduit is obtained. **c** The rolled conduit-extended left main coronary artery is re-implanted laterally to the aortic wall

Surgical correction was attempted in 8 patients (88.9%). Re-implantation (*n* = 6) (Fig. 3) and Takeuchi (*n* = 2) procedure were performed in these patients. One of the patients (11.1%) underwent VSD closure; two of the patients (22.2%) underwent surgical mitral valve repair at the same time, and one of them (11.1%) was implanted with a temporary pacemaker. During the surgery, the mean times of cardiopulmonary bypass (CPB) and aortic occlusion were 141 ± 31.5 and 105 ± 26.7 min, respectively. Blood transfusion was necessary for the patients who underwent surgical repair. One patient (11.1%) refused to undergo an operation.

At a mean follow up of 2 ± 0.8 years, all the patients were alive. One patient underwent another operation to deal with the dissociative steel wire after surgical repair, and the other 7 patients (77.8%) reported no complications. The patient who declined surgery was not followed up after discharge.

## Discussion

We present data on 9 patients diagnosed in childhood or adolescence with ALCAPA syndrome whose ages were between 5 and 16 years. Only one patient developed symptoms (apsychia), whereas other patients were asymptomatic, and there was no evident left ventricular dysfunction found in all the cases. ALCAPA syndrome mostly presents at the very beginning of life. Asymptomatic ALCAPA patients diagnosed in childhood or adolescence are even rarer and there are only a few cases reported [13].

ALCAPA also can be accompanied by other congenital heart diseases, but it is mostly an isolated malformation [5, 6, 14]. In these cases, one patient had a VSD and a dextropositioned aorta. Different imaging modalities were applied for the diagnosis of ALCAPA and they revealed the presence of collateral arteries. The main protective mechanism of ALCAPA patients is the development of collaterals which supply oxygenated blood from RCA to LCA [9, 10, 15]. When coronary steal syndrome arose or the collateral vessels were stenosed, those who survived the infantile period may present with symptoms [5, 6, 15]. ST changes and Q waves in the anterior and lateral leads are common ECG changes in ALCAPA patients [4–6]. In our series, ECG changes were observed in 7 (77.8%) patients. Echocardiography made the early diagnosis of ALCAPA in most of our patients and would play a more important role in the early diagnosis of asymptomatic adult-type ALCAPA in childhood or adolescence. The poor image quality in patients with difficult echo windows is the main limitation of echocardiography. It is also difficult to visualize the anomalous origin of LCA in the echocardiography. CT and CMR can directly provide visualization of the coronary artery anatomy with 3D reconstruction. Moreover, CMR could assess the size and function of biventricular and estimate abnormalities and shunts of the valve [16]. Coronary angiography could provide the detailed visualization of collaterals and quantify the left to right shunt.

In ALCAPA patients, MR is a common symptom that is observed early than even ventricular dysfunction [15, 17]. Our cases suggest that the accompaniment of dilated cardiomyopathy and MR is observed in asymptomatic children or adolescents with ALCAPA too. The timing of mitral valve repair at the time of ALCAPA remains controversial because mitral regurgitation degree may gradually decrease or remain stable in patients with preoperative mild and moderate mitral regurgitation without concomitant mitral valve repair, mitral valve repair or replacement is generally not necessary in this in this situation particularly in infants [18]. However, some authors advocate routine mitral valve repair at the of coronary artery revascularization because early postoperative cardiac output is improved and operative mortality is reduced [19]. We did not recommend concomitant mitral valve repair because mitral regurgitation in ALCAPA patients is related both to ischemic left ventricular dilatation with mitral annulus enlargement and to ischemic dysfunction of the papillary muscles and the mitral regurgitation may recover after the surgery [18]. Moreover performing complex mitral valve repair is time-consuming, thus increasing the ischemic time, which may be deleterious in patients with compromised myocardial function [20]. Due to the lifelong risk of ischaemia, ventricular arrhythmias, and sudden death due to cardiac arrest, surgery is the only definitive treatment of ALCAPA suggested even in asymptomatic adult patients [5, 6, 15]. However, the treatment for asymptomatic children or adolescents with ALCAPA was not mentioned. Considering the abnormal coronary system, dilated cardiomyopathy, and MR, early surgery is also necessary to recover normal circulation and prevent long-term myocardial ischaemia and fibrosis.

## Conclusion

With the development of imaging techniques, more asymptomatic adult-type ALCAPA patients could be diagnosed early in childhood or adolescence. Suspicion of ALCAPA should be raised when the echocardiograph shows a large RCA or a retrograde flow in the LCA. Early surgery is also necessary for asymptomatic children or adolescents with ALCAPA.

## Data Availability

All the data and material were restored in the database of West China Hospital.

## Abbreviations

ALCAPA: Anomalous origin of the left coronary artery from the pulmonary artery
PA: Pulmonary artery
LCA: Left coronary artery
LAD: Left anterior descending artery
RCA: Right coronary artery
PDA: Patent ductus arteriosus
LV: Left ventricle
VSD: Ventricular septal defect
CXR: Chest X-ray
CT: Cardiac Computed Tomography
MRI: Magnetic Resonance Imaging
CMR: Cardiovascular Magnetic Resonance
AHA: American Heart Association
